# Determination of Enantiomers of Deschloroketamine and Its Metabolites in Wistar Rat Brains

**DOI:** 10.1002/jssc.70503

**Published:** 2026-09-13

**Authors:** Natalie Paškanová, Vendulka Hrubá, Peter Frühauf, Bronislav Jurásek, Martin Kuchař, Pavel Řezanka, Michal Kohout

**Affiliations:** ^1^ Forensic Laboratory of Biologically Active Substances Department of Chemistry of Natural Compounds University of Chemistry and Technology Prague Prague 6 Czech Republic; ^2^ Department of Analytical Chemistry Faculty of Chemical Engineering University of Chemistry and Technology Prague Prague 6 Czech Republic; ^3^ Department of Analytical Chemistry University of Vienna Vienna Austria; ^4^ Department of Organic Chemistry University of Chemistry and Technology Prague Prague 6 Czech Republic

**Keywords:** brain tissue, capillary electrophoresis, chiral separation, deschloroketamine metabolites, supercritical fluid chromatography

## Abstract

Deschloroketamine (DXE) is a dissociative new psychoactive substance whose enantiomers and metabolites may exhibit distinct pharmacological and toxicological profiles. Reliable analytical methods enabling the chiral determination of DXE and its metabolites in biological matrices are thus essential. In this study, we evaluated supercritical fluid chromatography coupled to tandem mass spectrometry (SFC‑MS/MS) and capillary zone electrophoresis with ultraviolet detection (CZE‑UV) for the enantioselective analysis of DXE and its major metabolites in rat brain tissue. SFC using polysaccharide‑based chiral stationary phases provided baseline separation of the hydroxylated and demethylated metabolites, but severe peak distortion of DXE enantiomers prevented their quantification. Therefore, quantitative analysis was performed by CZE employing carboxymethyl‑β‑cyclodextrin as a chiral selector and a high‑sensitivity Z‑shaped detection cell. Analysis of brain tissue samples obtained following high‐dose administration revealed near equimolar concentrations of the DXE enantiomers and a higher concentration of (*R*)‐norDXE relative to (*S*)‐norDXE. In this particular case, the combined use of SFC‑MS/MS and CZE‑UV provided a practical workflow for the chiral profiling of DXE and its metabolites in complex biological matrices.

## Graphical abstract

Although supercritical fluid chromatography coupled to mass spectrometry efficiently resolved deschlorketamine and its metabolites, the parent drug must have been quantified by capillary zone electrophoresis.

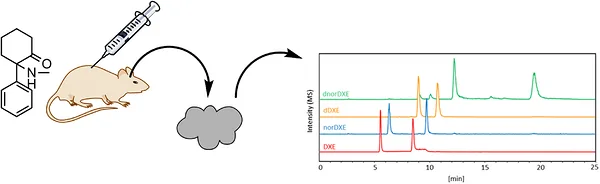

## 1 Introduction

Deschloroketamine (DXE) is a dissociative anesthetic classified as a new psychoactive substance (NPS) and structurally related to arylcyclohexylamines. Originally described in patent literature for potential antimicrobial and immunomodulatory effects [1, 2], DXE was later identified on the recreational drug market and analytically characterized as an emerging designer drug [3]. Pharmacological studies indicate that DXE acts as an *N*‐methyl‐D‐aspartate receptor antagonist and produces psychotomimetic and stimulant effects, with rapid penetration into the brain and higher brain‐to‐serum ratios than ketamine [4]. Repeated use has been associated with adverse physiological effects, including bladder inflammation, similar to those reported for other dissociative anesthetics [4, 5, 6].

The biotransformation of DXE primarily involves demethylation and hydroxylation, forming norDXE, *trans*‐dihydroDXE, and both *cis*/*trans*‐dihydronorDXE, as demonstrated in rat urine and serum analyses [4, 7, 8]. Additional metabolites, including hydroxylated, deaminated, and dehydrogenated derivatives, have been identified through non‑targeted screening approaches [7, 9, 10]. In a previous pharmacokinetic study, it was found that upon individual administration of each enantiomer, *R*‐DXE reached a higher maximal concentration in brain tissue, while the *S*‐enantiomer exhibited lower brain penetration [4]. Because DXE is consumed as a racemate, and experimental data suggest differences between enantiomers in behavioral potency, toxicity, and tissue distribution [4, 11], analytical methods capable of resolving and quantifying DXE enantiomers and their metabolites in biological matrices are essential.

Several analytical studies have addressed chiral separation of DXE or related NPS, typically using liquid chromatography with polysaccharide‐based chiral stationary phases (CSPs) [12, 13].

Nevertheless, ketamine remains the most extensively studied dissociative anesthetic from the perspective of enantioselective analysis, mainly due to the distinct pharmacological relevance of its enantiomers and chiral metabolites [14]. Consequently, several recent liquid chromatography‐tandem mass spectrometry (LC–MS/MS), supercritical fluid chromatography–MS, and capillary electrophoresis‐based methods have been developed for the stereoselective determination of ketamine, norketamine, hydroxynorketamine, and related metabolites in biological matrices [15, 16, 17]. To achieve sufficient chiral separation of ketamine and its metabolites, high‐performance LC relies on the combination of an achiral column with a CSP within a multicolumn switching system [17]. Other dissociative anesthetics, including DXE, can be resolved under polar organic mobile phase conditions [18], as well as using novel polysaccharide‐based CSPs [13]. However, existing methods generally focus on the parent compound and do not provide simultaneous enantioseparation of DXE together with its major metabolites in biological samples. Furthermore, the concentrations of DXE and metabolites in brain tissue are often low, imposing high demands on sensitivity and selectivity.

SFC‑MS/MS constitutes an effective approach for chiral analysis of psychoactive amines due to its efficiency, MS compatibility, and demonstrated performance for structurally related cathinones [19]. The effects of mobile‐phase composition, temperature, and CSP chemistry on enantioseparation of basic analytes are well documented [20, 21, 22]. Nevertheless, DXE presents a specific analytical challenge: pronounced peak distortion of its enantiomers has been observed under multiple SFC conditions, impairing quantification even when chromatographic resolution is achieved.

Capillary zone electrophoresis (CZE) with cyclodextrin modifiers represents a complementary analytical platform with different selectivity and has proven effective for the enantioseparation of chiral psychoactive amines [23, 24]. Optimization of electrolyte composition enables tuning of chiral interactions, and when combined with a high‑sensitivity ultraviolet (UV) detection cell, CZE is suitable for determining analytes present at low micromolar levels. Mathematical deconvolution using the Haarhoff–van der Linde (HVL) function additionally allows estimation of enantiomeric ratios in cases of partial separation [25].

In this study, we compare SFC‑MS/MS and CZE‑UV for the chiral determination of DXE and its main metabolites in rat brain tissue. We evaluate the extent to which each technique can resolve enantiomeric pairs, highlight methodological limitations—particularly the pronounced peak distortion of DXE in SFC—and establish a CZE‐based workflow enabling quantification of DXE and norDXE enantiomers. These two techniques offer certain complementarity under the applied conditions and can be used for stereoselective analysis of DXE in complex biological matrices.

## 2 Materials and Methods

### 2.1 Chemicals and Materials

**FIGURE 1:**
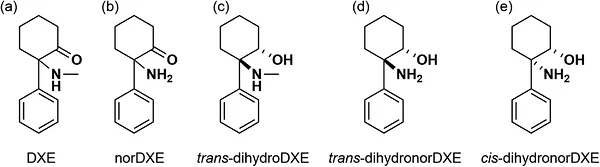
Structures of DXE (a) and its metabolites: norDXE (b), *trans*‐dihydroDXE (c), *trans*‐dihydronorDXE (d), and *cis*‐dihydronorDXE (e).

The following chemicals and solvents were used: acetonitrile (99.9%), methanol (99.9%), sodium chloride (99.5%), orthophosphoric acid (50%), ammonium bicarbonate, carboxymethylated β‐cyclodextrin sodium salt (CM‐β‐CD, average degree of substitution ∼3, 95%) (all Sigma‐Aldrich, Prague, Czech Republic), ultrapure water (Milli‐Q grade, Millipore, Molsheim, France), 1 mol·L^−1^ sodium hydroxide (Tripur, Merck, Darmstadt, Germany), hydrochloric acid (30%) (Suprapur, Merck, Darmstadt, Germany), sulfuric acid (96%) (Penta, Katovice, Czech Republic), sodium sulfate (Penta, Katovice, Czech Republic). Carbon dioxide 4.8 grade (99.998%) was obtained from Messer (Vienna, Austria), methanol LC‐MS Grade was purchased from VWR (Vienna, Austria), diethylamine (DEA, ≥ 99.5%), and formic acid (FA) were purchased from Sigma‐Aldrich (Schnelldorf, Germany). DXE and its derivatives norDXE, *trans*‐dihydroDXE, and *trans*/*cis*‐dihydronorDXE (Figure 1) in the form of hydrochloride salts were prepared previously [8]. Stock solutions were prepared in methanol and stored at −20°C.

### 2.2 Stock Solutions

DXE and its derivatives norDXE and dihydronorDXE were individually dissolved in water at a concentration of 20 mmol·L^−1^. The analyte solutions were further mixed and diluted with water to a final concentration of 1 mmol·L^−1^. For the standard addition method, mixtures of these analytes (Figure 1) at concentration ranges from 2 to 180 µmol·L^−1^ were also prepared.

### 2.3 Animals and Ethical Approval

All procedures involving animals in this study were conducted in accordance with EU Directive 86/609/EU and the guidelines of the National Committee for the Care and Use of Laboratory Animals. The experimental protocol was approved by the relevant ethics committee under the number 69371/ 2015‐MZE‐17214, and the research was carried out at the National Institute of Mental Health, Czech Republic.

### 2.4 Sample Preparation

The blank sample consisted of brain tissue without DXE. Two concentration levels were employed for the subcutaneous administration of DXE. The “high” concentration level was adjusted to CZE (B1 and B2; 2000 mg·kg^−1^ administration of DXE). The “low” concentration level corresponds to administration of DXE (B3; 10 mg·kg^−1^) and its enantiomers (*S*)‐DXE (B4; 10 mg·kg^−1^) and (*R*)‐DXE (B5; 10 mg·kg^−1^). For the CZE experiments, samples obtained after administration of a higher dose were used to ensure a sufficiently robust analytical response in the complex brain matrix and to enable reliable evaluation of enantiomeric composition. Lower‐dose samples were analyzed by SFC–MS/MS. The collection procedure of brain tissue has been published elsewhere [7].

Briefly, a sample preparation procedure based on salting‐out liquid‐liquid extraction was applied [11]. Brain tissue containing DXE (100 mg) was weighed into a microtube, ZY‐S type beads (200 mg) were added, and the mixture was suspended in ammonium bicarbonate (200 µL, 10 mmol·L^−1^) and acetonitrile (100 µL). The suspension was placed in a Bullet Blender homogenizer for 5 min (speed 6). The resulting homogeneous mixture was incubated in a freezer (–20°C) for 10 min. Following incubation, acetonitrile (300 µL) was added, and the resulting mixture was again homogenized using the Bullet Blender homogenizer for 5 min (speed 6). Subsequently, sodium chloride (20 mg) and sodium sulfate (100 mg) were added, and the mixture was again treated in the Bullet Blender homogenizer for 5 min. The homogeneous mixture was then centrifuged at 18 700 g for 5 min at 25°C. The formed supernatant (250 µL) was collected and evaporated to dryness. The evaporated sample was dissolved in 10% aqueous methanol (250 µL) and filtered through Mini‐spin PVDF filters (0.2 µm) by centrifugation at 18 700 g for 5 min at 25°C. The filtrate was transferred into a vial for analysis.

### 2.5 Equipment

The SFC analyses were carried out using the 1260 Infinity Analytical SFC System, which was coupled to a 6490 Triple Quadrupole Mass Spectrometer (both Agilent, Waldbronn, Germany) using an ESI interface. The MS parameters were optimized to maximize signal intensity of all analytes. The ESI spray voltage was set to 4000 V, gas flow 11 L·min^−1^, sheath gas temperature 400°C, sheath gas flow 12 L·min^−1^, gas temperature 250°C, and nebulizer gas pressure 35 psi. Multiple reaction monitoring transitions and the collision energies (CEs) were optimized experimentally; details are summarized in Table 1. Four different coated CSPs were used: Chiral ART Amylose‐C (CSP I) and Chiral ART Cellulose‐C (CSP III) (both YMC Europe GmbH, Dinslaken, Germany), Lux Amylose‐2 (CSP II) and Lux Cellulose‐2 (CSP IV) (both Phenomenex, Torrance, CA, USA). All columns had the same dimensions: 250 × 4.6 mm id, 5 µm.

**TABLE 1:** Selected parameters of the multiple reaction monitoring (MRM) method (CE = collision energy).

Analyte	Theoretical monoisotopic mass [M]	Experimentally measured mass (*m/z*)		CE (V)	Polarity
		Precursor ion MS1	Product ion MS2		
DXE	203.1	204	173; 91	20	Positive
		173	91; 79		
norDXE	189.1	190	173; 91	20	Positive
		173	91; 79		
*trans*‐dihydroDXE	205.2	206	157; 107; 91	20	Positive
		175	91; 79		
*trans/cis*‐dihydronorDXE	191.1	192	175; 157; 107; 91	30	Positive
		175	91; 79	20	

CZE separations were performed with an Agilent CZE instrument 7100 (Agilent 3D HPCE, Waldbronn, Germany) equipped with a UV‐visible (UV‐Vis) diode‐array detector. A bare fused‐silica capillary of 375/75 µm od/id and 58.5/50 cm total/effective length was obtained from Polymicro Technologies (Phoenix, AZ, USA).

Additional equipment used for sample preparation: Bullet Blender Storm BBY24M (Next Advance, Troy, NY, USA), Universal 320R centrifuge (Hettich, Germany), and CentriVap refrigerated concentrator (Labconco, Kansas City, MO, USA).

### 2.6 SFC‐MS/MS Method

Total runtime of the optimum method including column equilibration was 25 min. System parameters were set as follows: temperature 35°C, backpressure 150 bar, flow rate 2.5 mL·min^−1^, and the injection volume was 5 or 10 µL. The mobile phase was composed of supercritical carbon dioxide (scCO_2_) modified with methanol containing 0.1% DEA as an additive. The final gradient (scCO_2_/B) was: 0 min (95/5), 10 min (85/15), 10.01–21 min (85/15), 22 min (95/5), and 22.01–25 min (95/5). Chiral ART Amylose‐C (coated Amylose tris‐[3,5‐dimethylphenylcarbamate]) was employed for the chiral separation of the drug and its metabolites.

### 2.7 CZE Conditions

A new fused‐silica capillary was first rinsed with 1 mol·L^−1^ NaOH for 30 min, then with H_2_O for 30 min. Between the runs, the capillary was rinsed at 99.4 kPa first with 0.1 mol·L^−1^ NaOH for 2 min, then with H_2_O also for 2 min, and finally with the running buffer again for 2 min (for the capillary washing, a different buffer solution than for the subsequent analysis was used). The analytes were injected hydrodynamically at a pressure of 10 kPa for 20 s. Separations were performed at 20 kV (anode at the injection capillary end) with a voltage ramp time of 12 s. Detection using a flow cell and Z‐shaped flow cell was carried out at 200 nm with a bandwidth of 10 nm. The capillary was thermostated at 25°C during the analyses.

The background electrolyte (BGE) used consisted of 50 mmol·L^−1^ sodium phosphate buffer, pH 2.25 (50 mmol·L^−1^ orthophosphoric acid adjusted to appropriate pH with 1 mol·L^−1^ NaOH) and 25 mmol·L^−1^ CM‐β‐CD. CM‐β‐CD was selected based on earlier work with chiral amines, providing robust complexation and enantiodifferentiation under acidic BGE.

Quantitative validation included assessment of linearity, limits of detection (LODs), limits of quantification (LOQs), and injection repeatability using standard addition calibration in brain tissue extracts.

## 3 Results and Discussion

### 3.1 SFC‐MS/MS Method Development

First, we optimized the conditions for chiral separation of the analytes (for details see Supporting Information). Based on our previous experience with chiral resolution of cathinones of various polarities in SFC [19], we decided to use gradient elution with a mobile phase composed of scCO_2_ and methanol as a modifier. The initial conditions: scCO_2_/B (B = MeOH + 0.1% FA + 0.1% DEA); temperature 40°C; flow rate 4 mL·min^−1^ and back pressure 150 bar, were optimized (see Figure S1) by adjusting the modifier composition, temperature and flow rate [20, 21, 22], to reach the final optimum conditions given above (Chapter 2.6).

**FIGURE 2:**
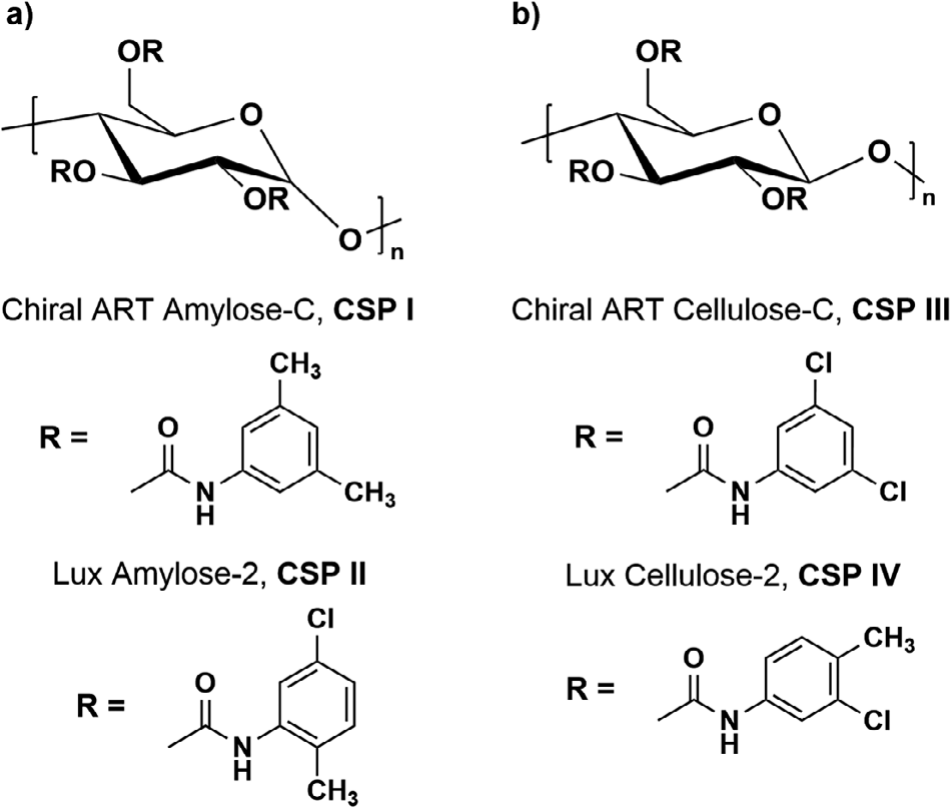
Structures of (a) amylose‐based chiral stationary phases and (b) cellulose‐based chiral stationary phases used in the study.

Initial screening of the CSPs (Figure 2) showed good results for **CSP I** and **CSP IV** (Table S1). **CSP II** and **CSP III** were much less effective for chiral resolution of the analytes. Comparing **CSP I** and **CSP III**, it can be assumed that the major effect is the polysaccharide backbone, which changes from amylose to cellulose. However, the superior chiral resolution observed on **CSP IV** in comparison to **CSP II** indicated that the polysaccharide backbone alone does not account for the difference.

DXE adopts a preferred conformation in which the phenyl unit is located in the axial position of the cyclohexane ring, enabling an intramolecular hydrogen bond between the amino group and the carbonyl oxygen [11]. Therefore, it is assumed that the chiral resolution of the enantiomers of the parent compound, and most probably also the formal metabolites, is primarily driven by hydrogen bonding and steric fit rather than π−π‐interactions. The substitution pattern in **CSP II** may result in steric repulsion between the phenyl unit and the analyte due to the methyl group neighboring the carbamoyl moiety. In the case of **CSP III**, the dichloro‐substitution reducing the electron density on the aromatic unit could reduce the hydrogen bond donation capacity of the carbamoyl nitrogen, which may result in a weak primary interaction. This assumption is supported by the short retention of all analytes.

The longest retention times were observed for **CSP IV**, which further supports the assumption that steric fit (3,4‐disubstituted aromatic unit) is very important for efficient chiral resolution of the studied analytes on polysaccharide‐based CSPs.

After achieving chiral resolution of the target compounds, we focused on mass detection. The obtained masses of precursor ions for the MS1 and product ions for MS2 are summarized in Table 1. For each analyte, the two most intense precursor ions and at least two product ions (for each precursor ion) were selected, and the optimal collision cell energy was set for them.

**FIGURE 3:**
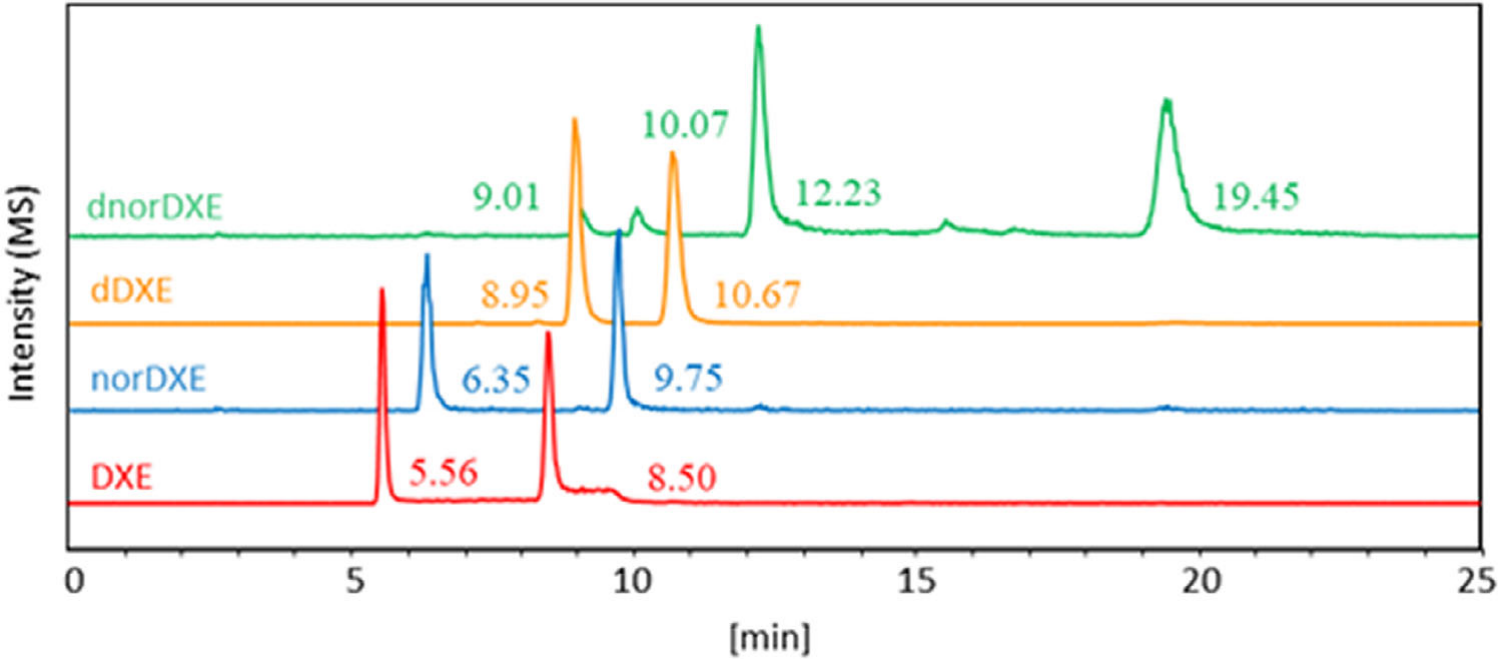
Chiral separation of deschloroketamine (DXE) and its metabolite standards under optimized conditions: ChiralArt Amylose‐C; scCO_2_/B (B = MeOH + 0.1% DEA); temperature 35°C; backpressure 150 bar; and flow rate 2.5 mL·min^−1^.

Employing the optimized method, we successfully resolved all analyte standards using Chiral ART Amylose‐C under gradient elution with a mobile phase composed of scCO_2_ and MeOH with DEA as an additive (Figure 3). The parent drug, however, exhibited tailing, which we were not able to successfully suppress by tuning the chromatographic conditions (variations in backpressure, temperature, and flow rate).

### 3.2 Determination of DXE and Its Metabolites in Brain Tissue by SFC‐MS/MS

**FIGURE 4:**
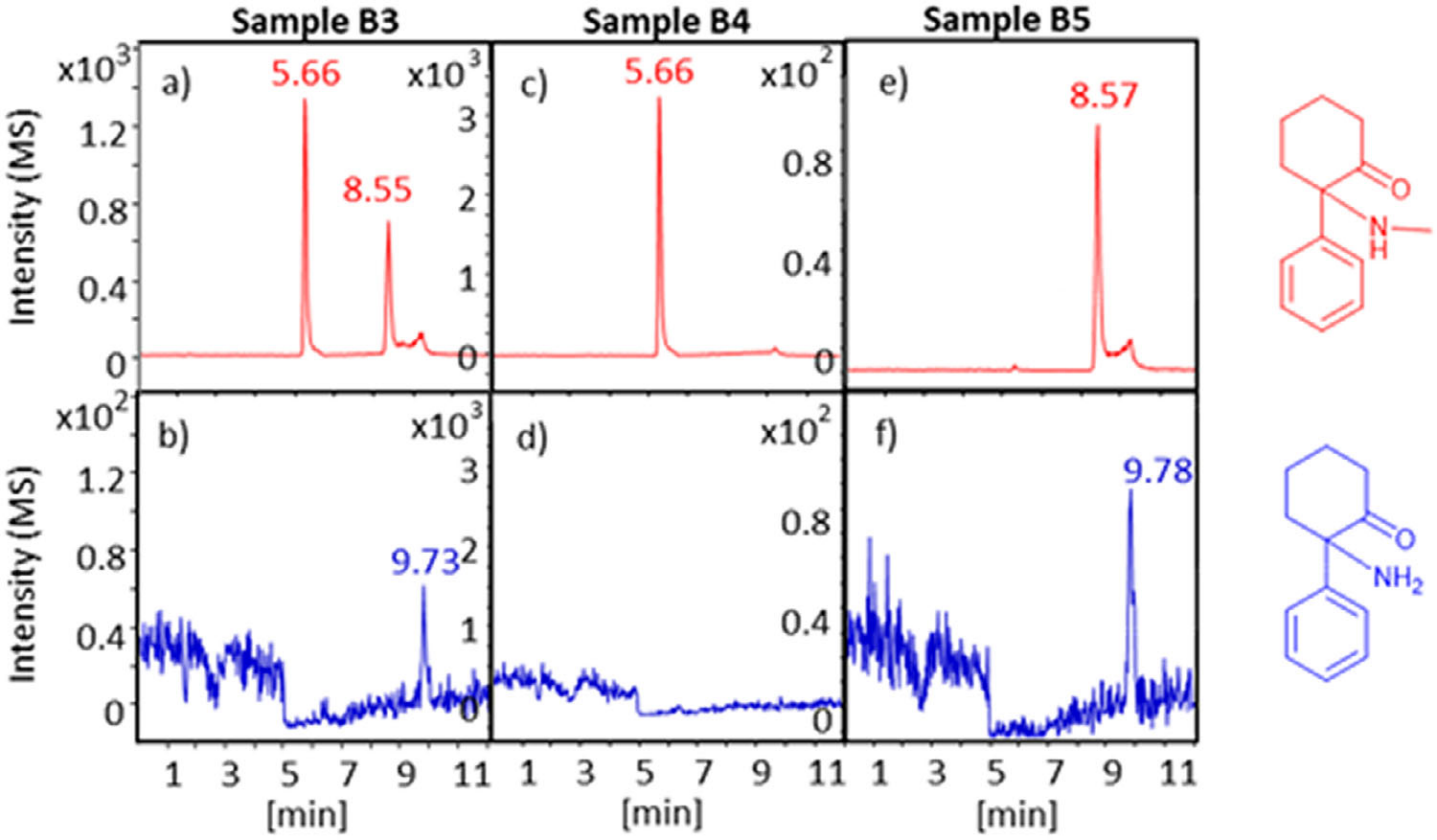
Supercritical fluid chromatography coupled to tandem mass spectrometry (SFC‑MS/MS) analysis of brain tissue samples prepared by salting out liquid‐liquid extraction (SALLE): deschloroketamine (DXE) and norDXE identified in the samples administered in racemic (a, b) and enantiomerically pure form (*S*)‐ (c, d) and (*R*)‐ (e, f) DXE.

First, we analyzed the samples obtained from previous behavioral studies using the SFC‐MS/MS method. The Wistar rats (B3, B4, and B5) received a low dose (10 mg·kg^−1^) of racemic DXE or the individual enantiomers [4]. In the brain tissue samples, we were able to detect DXE and its major metabolite, norDXE (Figure 4). In the samples from rats administered racemic DXE, we observed only (*R*)‐norDXE (Figure 4b). Analysis of the single enantiomer‐dosed samples (Figure 4c–f) suggested a lower concentration of (*S*)‐norDXE in brain tissue. Our findings supported previously published data obtained by an achiral method [4]. However, we were not able to verify our observation by quantification of DXE in the brain tissue because of a strong distortion of the peaks of DXE enantiomers (Figure 4a,c,e) that prevented an effective analysis and precluded reliable quantification of the drug.

### 3.3 Distortion of DXE Peaks in SFC‐MS/MS

**FIGURE 5:**
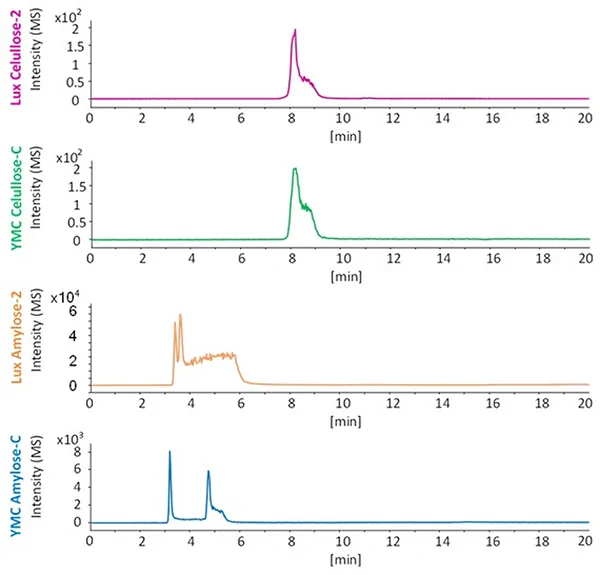
Comparison of the enantioseparation of deschloroketamine (DXE) by using all columns with the gradient of the mobile phase scCO_2_/methanol + 0.1% formic acid and 0.1% diethylamine (B). The used gradient (scCO_2_/B) was: 0 min (92/8), 12.5 min (82/18), 12.1–15 min (82/18), 15 min (92/8), and 15.01–20 min (92/8).

Peak distortion of DXE enantiomers was observed using all tested columns (Figure 5). Neither changing the temperature and ABPR pressure nor variation of mobile‐phase additives improved the situation. It is hypothesized that a chemical reaction between the parent drug and the mobile‐phase constituents occurs under SFC conditions. This hypothesis is supported by putative reaction products detected under various chromatographic conditions and identified by high‐resolution mass spectrometry. However, further investigation of this phenomenon is required to bring deeper insight into SFC chiral separation of DXE and potentially other analytes. These experiments are beyond the scope of this contribution, and a detailed study will be published elsewhere.

Given the limitations of chiral resolution of DXE in SFC, we focused on a different approach and developed a capillary zone electrophoresis‐based method.

### 3.4 Optimization of CZE Method

Because limits of quantitation using a UV detector typically exceed 1 mmol·L^−1^ for psychoactive amines, the following parameters were optimized in order to measure concentrations even several orders of magnitude below this threshold. For optimizing selected parameters, solutions of DXE at concentrations from 0.001 to 1 mmol·L^−1^ were used. Each measurement was performed in triplicate to optimize:

1. Type of the cell: a) flow cell and b) Z‐shaped flow cell;
2. Detection wavelength: a) 192 nm with the bandwidth of 4 nm, b) 200 nm with the bandwidth of 4 nm, c) 200 nm with the bandwidth of 10 nm, d) 200 nm with the bandwidth of 20 nm, and e) 210 nm with the bandwidth of 20 nm;
3. Injection parameters: a) 1.5 kPa for 5 s, b) 5 kPa for 10 s, and c) 10 kPa for 20 s;
4. pH of the BGE: a) 2.25 and b) 2.5.

**FIGURE 6:**
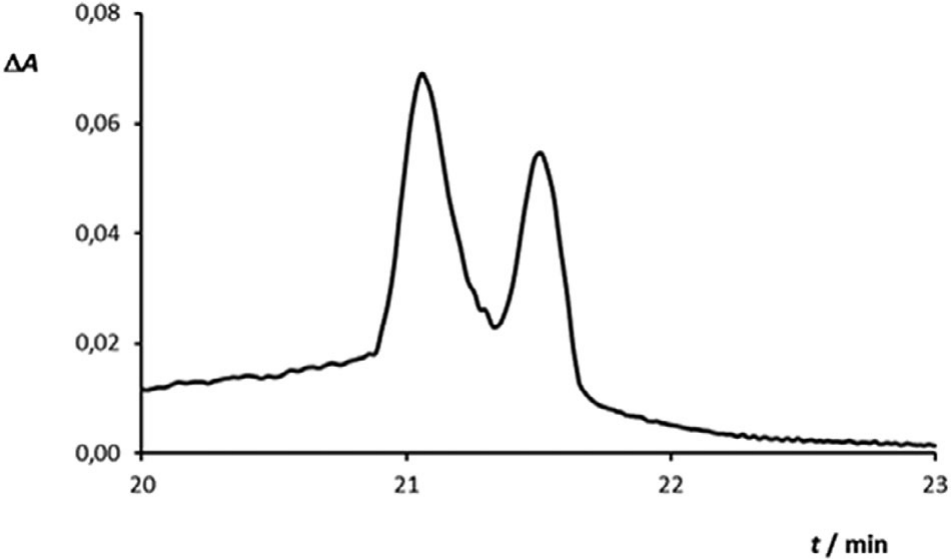
Electropherogram of the analyzed deschloroketamine (DXE) (0.01 mmol·L^−1^); fused‐silica capillary: od/id = 375/75 µm, total/effective length: 58.5/50.0 cm; background electrolyte (BGE): 50 mmol·L^−1^ phosphate buffer, pH 2.25, 25 mmol·L^−1^ CM‐β‐CD; voltage: 20 kV; temperature: 25°C; injection at 10 kPa for 20 s; Z‐shaped flow cell; detection at 200 nm with the bandwidth of 10 nm.

Electropherograms measured under different conditions (see 2.7) were evaluated in terms of DXE peak area. Of the studied parameters (type of cell, used wavelength and injection parameters), the analysis with the Z‐shaped flow cell using detection at 200 nm with the bandwidth of 10 nm, injection at 10 kPa for 20 s and pH of the BGE 2.25 proved to be the most suitable (Tables S2–S5). The electropherogram obtained by analyzing DXE (0.01 mmol·L^−1^) using the most suitable parameters is shown in Figure 6. This concentration is more than 100‐fold lower than usually used in CZE.

### 3.5 Determination of DXE and Its Metabolites in Brain Tissue by CZE

**FIGURE 7:**
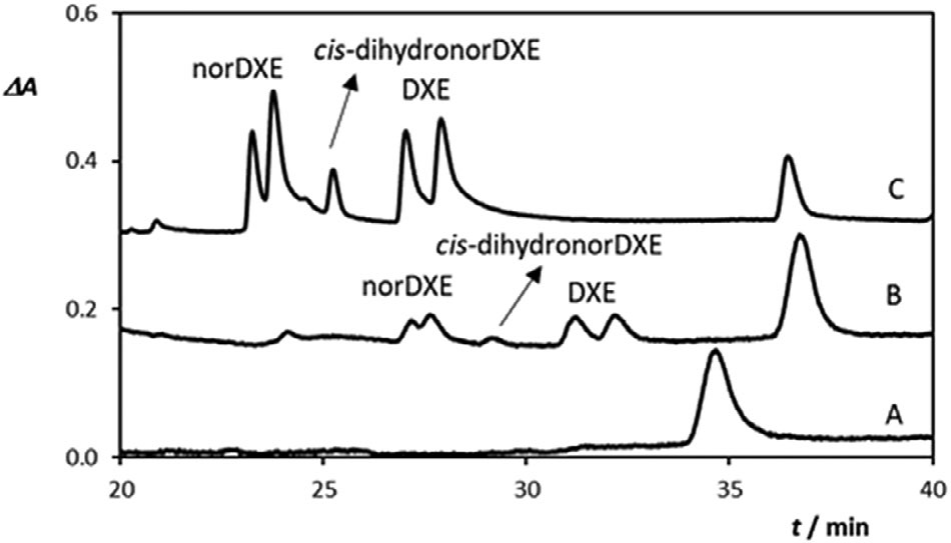
Electropherograms of the analyzed: blank sample (A), B1 (B) and B1 with the addition of deschloroketamine (DXE) and its metabolites of the resulting concentrations 0.04 mmol·L^−1^ (C); fused‐silica capillary: od/id = 375/75 µm, total/effective length: 58.5/50.0 cm; background electrolyte (BGE): 50 mmol·L^−1^ phosphate buffer, pH 2.25, 25 mmol·L^−1^ CM‐β‐CD; voltage: 20 kV; temperature: 25°C; injection at 10 kPa for 20 s; Z‐shaped flow cell; detection at 200 nm with the bandwidth of 10 nm.

The areas in the electropherograms obtained from the samples (see 2.7) analyses corresponding to the relevant analytes were evaluated using the standard addition method in the range of 2–180 µmol·L^−1^ (see Figure 7 for electropherograms of blank sample, B1 without and with the addition of DXE and its metabolites). As shown in Figure 7, the addition of DXE and its metabolites decreased their migration time, while the migration time of the impurity (around 35.5 min) did not change. This behavior is likely attributable to a lower proportion of analyte molecules forming inclusion complexes with the cyclodextrin selector, which results in a higher effective positive charge density, and, consequently, greater electrophoretic mobility.

Quantitative performance of the method was evaluated using the standard addition approach in brain tissue extracts to account for matrix effects in brain tissue. Good linearity was obtained over the investigated concentration range (2–180 µmol·L^−^^1^), with correlation coefficients (*R* ^2^) exceeding 0.99 for all analytes (Table S5). Method sensitivity was characterized by limits of detection and quantification of 3.4–6.2 and 10.2–18.9 µmol·L^−^^1^, respectively. Precision assessed from repeated injections (*n* = 3) showed relative standard deviations below 4.9%. These validation parameters confirm that the developed CZE method is suitable for quantitative analysis of DXE and its metabolites in rat brain tissue.

As shown in Figure 7, sufficient chiral resolution of DXE was achieved, and allowed us to determine the relative ratio of enantiomers (Table 2), which is consistent with near‐equimolar presence of both enantiomers. The resolution of the major metabolite norDXE was also achieved. Although the enantiomers were not baseline resolved, we were able to perform mathematical separation of the peaks using the HVL function, which enabled the determination of the relative ratio of enantiomers (please see Supporting Information for the calculation procedure). Consistent with the SFC‐determined relative ratio, (*R*)‐norDXE was more abundant than (*S*)‐norDXE for brains at high concentration levels.

**TABLE 2:** Concentrations of deschloroketamine (DXE) and its metabolites in rat brain tissue (µg·g^−^^1^, mean ± SD, three injections per sample).

Sample	(*R*)‐DXE (µg·g^−^^1^)	(*S*)‐DXE (µg·g^−^^1^)	dihydronorDXE (µg·g^−^^1^)	(*S*)‐norDXE (µg·g^−^^1^)	(*R*)‐norDXE (µg·g^−^^1^)
B1	0.10 ± 0.07	0.10 ± 0.07	0.47 ± 0.17	0.14 ± 0.07	0.29 ± 0.14
B2	0.16 ± 0.04	0.17 ± 0.05	0.32 ± 0.14	0.10 ± 0.07	0.20 ± 0.15

### 3.6 Discussion

As shown above for SFC‐MS/MS analysis, a rat (single sample) that was administered the racemic DXE at the low concentration (10 mg/kg) was found to have a higher relative concentration of (*R*)‐norDXE in the brain. This observation could be explained by (i) preferential transport of the metabolite into the brain, (ii) preferential metabolism of (*R*)‐DXE in the brain, (iii) coincidental anomaly with no biological significance. To exclude case (iii), it is necessary to consider our previous study [4], in which we described different levels of drugs in rat serum and brain on administration of single enantiomers, which suggested possible active transport of DXE into the brain. Therefore, it is reasonable to assume that one of the remaining possibilities, (i) or (ii), may account for the observation. To test this hypothesis, we attempted the chiral resolution of the parent drug; however, we were not able to diminish its tailing, which precluded reliable quantification. Therefore, we developed the CZE method using a different series of rat brain samples. For a direct comparison of both SFC and CZE methods, see Table S6.

Since CZE with UV detection typically reaches higher detection limits than MS, we used brain samples collected after the administration of a relatively high dose of the drug (2000 mg·kg^−1^). The use of higher‐dose samples in the CZE experiments should be understood in a methodological context, as it allowed reliable assessment of enantiomeric ratios despite matrix complexity. In contrast, SFC–MS/MS was employed for the analysis of physiologically more relevant low‐dose samples. This highlights the complementary roles of both techniques within the proposed analytical workflow. Despite this dose difference between SFC and CZE samples, we documented a higher brain concentration of (*R*)‐DXE compared with (*S*)‐DXE.

As already mentioned above, relative differences in the concentrations of (*R*)‐DXE and (*S*)‐DXE were observed in brain tissue and serum after the administration of single enantiomers. The maximum concentrations of the major metabolites were found 1 h after administration, with higher values for (*R*)‐norDXE than for *S*‐ norDXE [4]. Despite the limited sample set used in this study, our findings for the administered racemic drug provide additional evidence that DXE enantiomers and, particularly, their metabolites may undergo stereoselective distribution or metabolism in the brain.

## 4 Conclusions

This study compared SFC‑MS/MS and CZE‑UV for the enantioselective analysis of DXE and its major metabolites in rat brain tissue. SFC‑MS/MS provided baseline enantioseparation for all hydroxylated and demethylated metabolites, demonstrating its suitability for chiral profiling of DXE biotransformation products. However, pronounced peak distortion of DXE enantiomers was consistently observed under multiple chromatographic conditions, preventing reliable quantification of the parent compound.

CZE‑UV, employing carboxymethyl‑β‑cyclodextrin as a chiral selector, offered a practical alternative for quantitative analysis. Under the optimized conditions, the method enabled separation of DXE enantiomers and mathematical deconvolution of partially resolved norDXE enantiomers using the Haarhoff–van der Linde function. Analysis of high‑dose brain samples revealed near‐equimolar concentrations of both DXE enantiomers and a higher concentration of (*R*)‐norDXE compared to (*S*)‐norDXE.

In this particular case, the combined application of SFC‑MS/MS and CZE‑UV provides a complementary analytical workflow. SFC‑MS/MS is well suited for sensitive detection and chiral resolution of DXE metabolites, while CZE‑UV enables the quantification of DXE and norDXE at high concentration levels when SFC is limited by peak‑shape distortions. This integrated approach expands the analytical options for studying the stereoselective distribution and metabolism of DXE in complex biological matrices.

## Author Contributions

**Natalie Paškanová**: methodology, investigation, Writing – original draft, and visualization; **Vendulka Hrubá**: investigation; **Peter Frühauf**: investigation, methodology, and supervision; **Bronislav Jurásek**: conceptualization and writing – review & editing; **Martin Kuchař**: project administration, resources, supervision, and funding acquisition; **Pavel Řezanka**: investigation, supervision, and writing – review & editing; **Michal Kohout**: conceptualization, resources, supervision, and writing – review & editing.

## Conflicts of Interest

The authors declare no conflicts of interest.

## Funding

This research was supported by the Ministry of Interior of the Czech Republic (grant No. VK01010212).

## Supporting information




**Supporting File**: jssc70503‐sup‐0001‐SuppMat.docx.

## Data Availability

Raw data are available from the authors upon legitimate interest.
