# Supplementary material for: Determination of Enantiomers of Deschloroketamine and Its Metabolites in Wistar Rat Brains
Source: J Sep Sci. 2026 Sep 13;49(9):e70503. doi: 10.1002/jssc.70503 (PMC13572844; doi:10.1002/jssc.70503)
Supplement: Supplementary file 1 [file JSSC-49-e70503-s001.docx]

*Electronic Supplementary Information*

Determination of Enantiomers of Deschloroketamine and Its Metabolites in Wistar rat Brains

Natalie Paškanová ^1,*^, Vendulka Hrubá ^2^, Peter Frühauf ^3^, Bronislav Jurásek ^1^, Martin Kuchař ^1^, Pavel Řezanka ^2^, Michal Kohout ^4,*^

^1^	Forensic Laboratory of Biologically Active Substances, Department of Chemistry of Natural Compounds, University of Chemistry and Technology, Prague, Technická 3, 166 28 Prague 6, Czech Republic;

^2^	Department of Analytical Chemistry, Faculty of Chemical Engineering, University of Chemistry and Technology, Prague, Technická 5, 166 28 Prague 6, Czech Republic;

^3^	Department of Analytical Chemistry, University of Vienna, Währinger Strasse 38, A-1090 Vienna, Austria

^4^	Deparment of Organic Chemistry, University of Chemistry and Technology, Prague, Technická 5, 166 28 Prague 6, Czech Republic;

*****	Correspondence:

This ESI file comprise information on optimization of chiral separation of the studied analytes in supercritical fluid chromatography (SFC) and capillary zone electrophoresis (CZE).

Composition of mobile phase, elution mode and selection of stationary phase for SFC-MS/MS Method

Four CSPs differing in the polysaccharide backbone and its chemical modification were compared: Chiral Art Amylose-C (coated Amylose tris-[3,5-dimethylphenylcarbamate]), Chiral Art Cellulose-C (coated Cellulose tris-[3,5-dimethylphenylcarbamate]) Lux Amylose-2 (coated Amylose tris[5-chloro-2-methylphenylcarbamate]), Lux Cellulose-2 (coated Cellulose tris[3-chloro-4-methylphenylcarbamate]). Pre-optimized conditions were as follows: gradient elution CO_2_/B (B = MeOH + 0.1% formic acid + 0.1% diethylamine); temperature 40 °C; flow rate 4 mL·min^-1^ and back pressure 150 bar. Lowering the concentration of additives led to increasing retention times and worse chiral resolution.

Only one column, Chiral ART Amylose-C provided enantioseparation of all analytes (Table SI1). Lux Cellulose-2 provided better resolution for *N*-desmethyl analytes. On the other hand, no enantioseparation of DXE and *trans*-dihydroDXE was obtained. The shortest retention time and lowest resolution were observed on Chiral ART Cellulose-C.

Temperature effects SFC-MS/MS

To increase the chiral resolution of the target analytes, we also investigated the effect of temperature. The temperature study was carried out with a temperature step of 5 °C from 20 to 50 °C (Figure SI1). The comparison of the chromatograms of two structurally very similar substances clearly documents that even a slight change in the structure of the analyte has a significant influence on the chromatographic properties. Lowering the column temperature increased retention like Hofstetter observed for similar substances.^S1^ Shifting in minutes was observed for norDXE; much less significant shifting was observed in case *trans*-dihydroDXE.

Table SI1. Chromatographic parameters for all tested columns (non-optimized gradient of mobile phase; flow rate: 4 mL min^-1^; temperature: 40 °C, back pressure: 150 bar)

| Analytes |  | Chiral ART Amylose-C | | | |  | Lux Amylose-2 | | | |  | Chiral ART Cellulose-C | | | |  | Lux Cellulose-2 | | | |
| --- | --- | --- | --- | --- | --- | --- | --- | --- | --- | --- | --- | --- | --- | --- | --- | --- | --- | --- | --- | --- |
|  |  | *t_1_* | *k_1_* | *α* | *R* |  | *t_1_* | *k_1_* | *α* | *R_s_* |  | *t_1_* | *k_1_* | *α* | *R* |  | *t_1_* | *k_1_* | *α* | *R* |
| DXE |  | 3.21 | 3.52 | 1.62 | **1.20** |  | 3.37 | 3.21 | 1.08 | 0.75 |  | 2.37 | 2.33 | - | - |  | 8.19 | 9.93 | - | - |
| norDXE |  | 3.37 | 3.75 | 1.73 | 2.05 |  | 3.09 | 2.87 | 1.20 | 0.71 |  | 2.43 | 2.42 | 1.00 | 1.02 |  | 8.64 | 10.51 | 1.27 | **2.13** |
| *trans*-dihydroDXE |  | 3.37 | 3.75 | 1.74 | **2.98** |  | 5.66 | 6.08 | 2.12 | 1.83 |  | 3.25 | 3.58 | 1.10 | 0.30 |  | 10.92 | 13.56 | 1.02 | 0.35 |
| *trans*-dihydronorDXE |  | 5.62 | 6.91 | 1.04 | 0.54 |  | 5.18 | 5.48 | - | - |  | 4.37 | 5.16 | - | - |  | 10.12 | 12.50 | 1.15 | **2.13** |
| *cis*-dihydronorDXE |  | 7.47 | 9.52 | 1.49 | 2.74 |  | 5.96 | 6.45 | - | - |  | 4.68 | 5.59 | - | - |  | 13.08 | 16.44 | 1.47 | **4.89** |

„-„ means no detection

Figure SI1. Effects of temperature variation on enantioseparation of analytes DXE, norDXE and *trans*-dihydroDXE and dihydronorDXE on column Lux Cellulose-2.

Optimization of CZE Method

To compare individual parameters, signal-to-noise ratios (*S*/*N*) were evaluated. The results of the optimization procedures are presented in Tables SI2-5.

Table SI2. *S/N* ratios different cells; Detection wavelength: 200 nm with the bandwidth of 10 nm; DXE (0.01 mmol·l^−1^); fused-silica capillary: od/id = 375/75 μm, total/effective length: 58.5/50.0 cm; BGE: 50 mmol·l^−1^ phosphate buffer, pH 2.25, 25 mmol·l^−1^ CM-β-CD; voltage: 20 kV; temperature: 25 °C; injection at 10 kPa for 20 s.

| Cell | *S/N* | RSD (%) |
| --- | --- | --- |
| flow cell | 1595 | 2.1 |
| Z shaped flow cell | 8623 | 2.2 |

Table SI3. *S/N* ratios for different ranges of wavelengths and bandwidths; DXE (0.01 mmol·l^−1^); fused-silica capillary: od/id = 375/75 μm, total/effective length: 58.5/50.0 cm; BGE: 50 mmol·l^−1^ phosphate buffer, pH 2.25, 25 mmol·l^−1^ CM-β-CD; voltage: 20 kV; temperature: 25 °C; injection at 10 kPa for 20 s; Z shaped flow cell.

| Detection wavelength (bandwidth) | *S/N* | RSD (%) |
| --- | --- | --- |
| 192 nm (4 nm) | 2669 | 1.8 |
| 200 nm (4 nm) | 4660 | 2.4 |
| 200 nm (10 nm) | 8623 | 2.2 |
| 200 nm (20 nm) | 6972 | 1.7 |
| 210 nm (20 nm) | 5473 | 1.1 |

Table SI4. *S/N* ratios for different injection parameters (time and pressure); DXE (0.01 mmol·l^−1^); fused-silica capillary: od/id = 375/75 μm, total/effective length: 58.5/50.0 cm; BGE: 50 mmol·l^−1^ phosphate buffer, pH 2.25, 25 mmol·l^−1^ CM-β-CD; voltage: 20 kV; temperature: 25 °C; Z shaped flow cell detection at 200 nm with the bandwidth of 10 nm.

| Pressure / Time | *S/N* | RSD (%) |
| --- | --- | --- |
| 1.5 kPa / 5 s | 1240 | 2.3 |
| 5 kPa / 10 s | 3891 | 1.9 |
| 10 kPa / 20 s | 8623 | 2.2 |

The evaluation of the electropherograms obtained by the analysis at different pH showed that it is better to use pH 2.25 (the resolution for DXE was 1.4 and for norDXE was 0.9) than pH 2.5 (the resolution for DXE was 1.1 and for norDXE was 0.7).

*Determination* of the content of DXE and its metabolites (cis/trans-dihydronorDXE and *norDXE) in rat brain tissue*

The concentrations of the monitored analytes were determined using the method of standard additions in the range of 2–180 μmol·l⁻¹ (see Section 3.4 of the manuscript). Peak areas were obtained from the electropherograms using the Haarhoff–van der Linde (HVL) function*. The concentrations of the respective analytes were then calculated according to the standard procedure used for calibration‑curve‑based quantification.

* Because the relevant peaks in the electropherograms were not baseline‑separated, the experimentally acquired peak shapes were fitted using the HVL function (Eq. 1) in OriginPro 2018b (OriginLab Corporation, USA). The parameter a₀, corresponding to the integrated peak area, was subsequently used for quantitative calculations.

$\text{HVL}_{\text{δ}}(t;a_{0},a_{1},a_{2},a_{3})=\frac{\frac{a_{0}\cdot a_{2}}{a_{2}\cdot a_{3\text{δ}}\sqrt{2\pi}}\cdot e^{-0.5\cdot\left[\left(t-a_{1}\right)/a_{2}\right]^{2}}}{\frac{1}{e^{a_{3\text{δ}}}-1}+0.5\cdot\left[1+\text{erf}\left(\frac{t-a_{1}}{\sqrt{2}\cdot a{}_{2}}\right)\right]}$ (Eq. 1)

The analytical performance of the CZE method was further evaluated. All analytes exhibited excellent linearity (R² > 0.99). Linear range, limits of detection and quantification were estimated based on the calibration data. The obtained parameters are summarized in Table SI5.

Table SI5. Analytical performance characteristics of the CZE method.

| Analyte | Linear range (μmol·l⁻¹) | LOD (μmol·l⁻¹) | LOQ (μmol·l⁻¹) |
| --- | --- | --- | --- |
| DXE | 17.0–180 | 5.6 | 17.0 |
| dihydronorDXE | 18.9–180 | 6.2 | 18.9 |
| norDXE | 10.2–180 | 3.4 | 10.2 |

Comparison of SFC and CZE methods

Table SI6. Comparison of SFC and CZE methods used in the study.

| **Parameter** | | **SFC-MS/MS** | **CZE-UV** |
| --- | --- | --- | --- |
| Detection principle | | Tandem mass spectrometry | UV detection |
| Introduction of chirality | | Polysaccharide-based chiral stationary phase | CM-β-CD added to BGE |
| Chiral selector | | Chiral ART Amylose-C | Carboxymethyl-β-cyclodextrin |
| Enantioseparation | DXE | Achieved, but severe peak distortion observed | Successful separation suitable for quantification |
|  | norDXE | Baseline/near-baseline separation | Partial separation with HVL deconvolution |
|  | trans-dihydroDXE | Baseline separation | Not evaluated / not detected |
|  | dihydronorDXE | Baseline separation | Not evaluated / not detected |
| Suitability for DXE quantification | | Limited due to peak distortion | Suitable |
| Suitability for metabolite profiling | | Excellent sensitivity/selectivity | Limited |
| Typical analysis time | | ~25 min | ~35–40 min |
| Main advantage | | Sensitive MS detection and broad metabolite coverage | Robust quantification of DXE enantiomers |
| Main limitation | | Distortion/tailing of DXE peaks | Lower sensitivity compared to MS |

*References*

S1	Hofstetter, R. K., Potlitz, F., Schulig, L., Kim, S., Hasan, M., Link, A. Subcritical fluid chromatography at sub-ambient temperatures for the chiral resolution of ketamine metabolites with rapid-onset antidepressant effects. *Molecules*, 2019, *24*(10), 1927. https://doi.org/10.3390/molecules24101927
